# Value of ultrasound-guided aspiration of hip arthroplasties performed in an orthopedic clinic by orthopedic surgeons

**DOI:** 10.5194/jbji-6-393-2021

**Published:** 2021-11-10

**Authors:** Holly Duck, Suzanne Tanner, Debra Zillmer, Douglas Osmon, Kevin Perry

**Affiliations:** 1 Department of Orthopedic Surgery, Mayo Clinic, Rochester MN, USA; 2 Department of Infectious Diseases, Mayo Clinic, Rochester MN, USA

## Abstract

**Background**: Total joint arthroplasties continue to increase as do periprosthetic joint infections (PJIs). Ultrasound-guided aspiration can yield useful synovial fluid for analysis while avoiding radiation exposure. This study presents a high-yield, ultrasound-guided technique with analysis of aspiration results. **Methods**: All consecutive ultrasound-guided aspirations of hip arthroplasties performed from May 2016 through to April 2019 were retrospectively reviewed. Patient demographic information, component specifics, presence of draining sinus, and inflammatory markers were recorded. Results of aspiration including volume, appearance, lavage use, synovial fluid differential leukocyte count, synovial neutrophil percent, and culture results were recorded. Surgical results, specimen cultures, and surgeon description of purulence were recorded. Aspiration results were compared to the surgical specimen results in all patients who underwent reoperations. **Results**: Review of 349 hip aspirations demonstrated accuracy of 87 %, sensitivity of 83 %, specificity of 89 %, positive predictive value of 79 %, and negative predictive value 91 %. Surgical and aspiration cultures matched in 81 % of cases. Bloody aspirates and aspirates obtained after lavage had less accuracy at 69 % and 60 %, respectively. Specificity was 100 % for cultures obtained with lavage and 91 % for bloody aspirates. Synovial leukocyte count and neutrophil percentage was obtained in 85 % of aspirations, and cultures were obtained in 98 % of aspirates. Contamination rate was 2 %. **Conclusion**: Ultrasound-guided aspirations aid in the diagnosis of PJI. The use of lavage to obtain fluid is helpful when aspiration cultures are positive. Bloody aspirates are less accurate but have high specificity. A low contamination rate and 88 % accuracy results with this meticulous technique.

## 1 Introduction

Total joint arthroplasties continue to increase so that over 600 000 total hip arthroplasties will be performed in the United States annually by 2030 (Kurtz et al., 2007, 2014; Sloan et al., 2018). Periprosthetic joint infection (PJI) is a potentially devastating complication of a total hip arthroplasty with a rate of 0.5 % to 1 % of primary hip arthroplasties. Aspiration of the affected joint to obtain synovial fluid is the cornerstone for identifying the presence of infection and guide if revision is needed (Perry and Hanssen, 2017). Analysis of synovial fluid also fosters optimization of antimicrobial therapy through selection of the most narrow and active agent if likely causative organisms are identified by culture (Beam and Osmon, 2018), aids in surgical planning such as determining if all foreign material should be removed (Barrack and Harris, 1993), and aids in managing patient expectations (Isern-Kebschull et al., 2019).

Traditionally, aspirations of hip arthroplasties have been performed in fluoroscopy suites. Ultrasound use for musculoskeletal applications is expanding, including for injections (Balog et al., 2017; Henne et al., 2021; Li et al., 2018; Lynch et al., 2019). As of this writing, there is only one study in which aspirations for diagnosis of prosthetic hip infections are compared with fluoroscopic versus ultrasound guidance. This study was limited to 52 patients (Randelli et al., 2018). Ultrasound usage was less expensive and fluid studies were slightly more accurate (Randelli et al., 2018). Use of ultrasound, rather than fluoroscopy, may be more convenient for patients, eliminates the need for special suites, avoids exposing patients and staff to radiation, and may result in greater patient satisfaction with less pain (Byrd et al., 2014). Fluid collections in soft tissues may be identified and aspirated (Craig, 2013), which is not feasible with fluoroscopy. Another recent study describes using ultrasound guidance to biopsy tissue in suspected infectious hip arthroplasties due to a dry tap rate of 37 % (Sconfienza, 2021). This required use of a Tru-Cut needle with some discordant results. A landmark-guided technique has been described by Li et al. (2021), but no fluid (i.e., dry tap) was obtained in 45 % of aspirations.

This study was performed to

1. present a high-yield, ultrasound-guided technique for aspirating synovial fluid from hip arthroplasties without need of biopsy and avoidance of dry taps;
2. determine yield and accuracy of aspirated fluid in detection of PJI using this technique; and
3. analyze effects of body mass index (BMI), presence of draining sinus or antibiotic cement, use of saline lavage, and presence of blood on obtained fluid.

## 2 Materials and methods

All consecutive ultrasound-guided aspirations of hip arthroplasties performed in an orthopedic clinic from May 2016 through to April 2019 were included. Our institutional review board (IRB) deemed this study exempt from review. Power analysis assuming an infection rate of 15 % (based on review of 100 preliminary records) required sample size of 220 patients. Aspirations were performed in patients with painful primary total hip arthroplasties, revision arthroplasties, and hemiarthroplasties. Hips with temporary metal spacers with antibiotic cement were aspirated to determine if prior infection had been eradicated. Aspirations in the settings of native hips, resection arthroplasties, cement-only temporary spacers, and femoral head resurfacing were excluded since the aspiration technique used varies from that described in this study. Aspirations in patients who did not permit record review consent were excluded. Patient demographic information that was recorded included age, sex, and body mass index. Extremity involved, position of the components (dislocated or not), presence of draining sinus, and presence of antibiotic cement were identified. Serum complete blood count (CBC), C-reactive protein (CRP), and erythrocyte sedimentation rate (ESR) were recorded. Fluid appearance, volume, and use of saline lavage were tabulated. Synovial fluid differential leukocyte count and culture results were recorded. For patients who underwent a subsequent operation by February 2020, the surgical tissue pathology and culture findings were recorded. Operative reports and clinic notes were reviewed to determine if purulence was seen and if the treating surgeon managed the hip arthroplasty as if infected. Using International Consensus Meeting (ICM) 2018 criteria (Parvizi et al., 2018), arthroplasties were categorized as infected, not infected, inconclusive, or insufficient data. The value of the aspiration culture results alone to predict infections was determined by calculating accuracy, sensitivity, specificity, positive predictive value (PPV), and negative predictive value (NPV), each with 95 % confidence intervals compared to ICM criteria. For each aspiration, it was determined whether the culture results matched the surgical specimen result, and instances of contaminants were determined.

**Figure 1:**
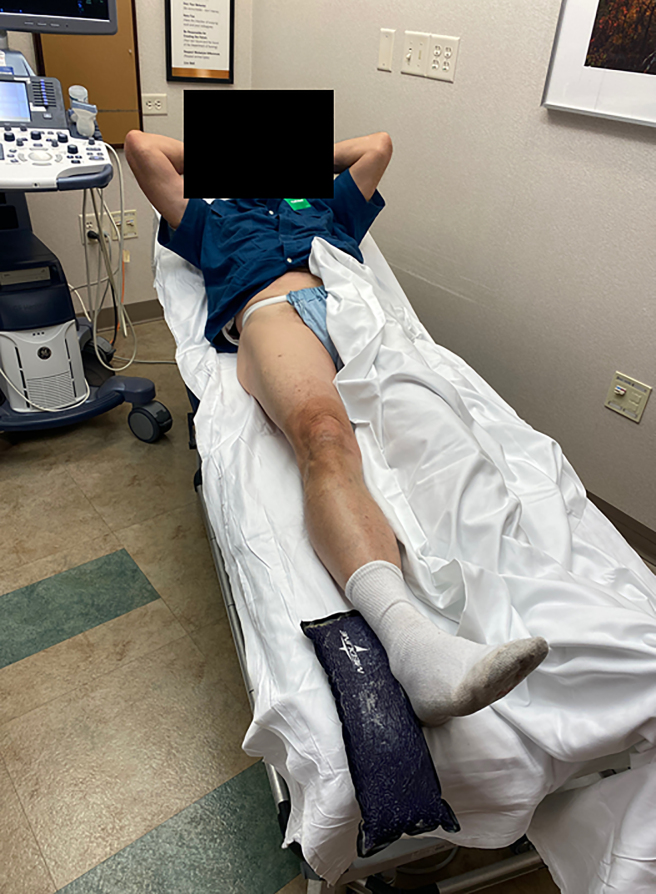
Patient positioning.

## 3 Technique

The technique was performed with the patient in supine position (Fig. 1).

**Figure 2:**
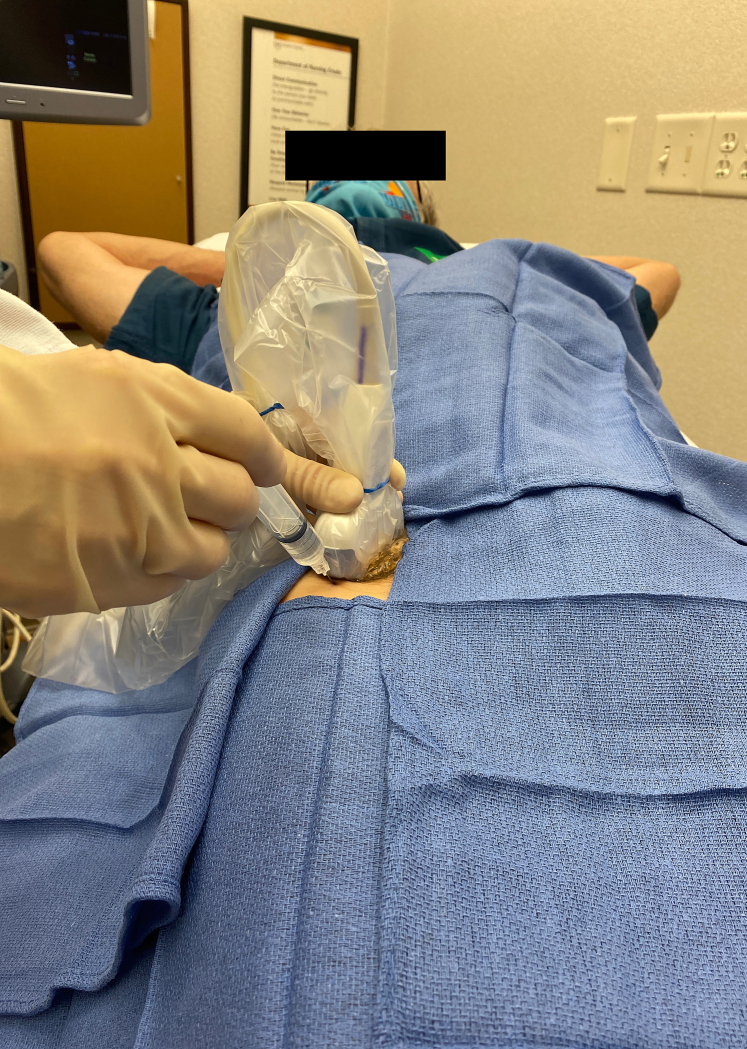
Transducer position.

A low-frequency curvilinear (4 MHz usually; 3 MHz for obese patients) transducer was used. To identify the neck of the prosthesis, the anterior femoral shaft was identified with the transducer placed longitudinally (i.e., from proximal to distal) in the sagittal plan. The transducer was moved proximally to the level of the greater trochanter. The transducer was then angled 45 ${}^{\circ }$ toward the groin, so it was parallel with the neck of the prosthesis, distal to the inguinal crease (Fig. 2).

**Figure 3:**
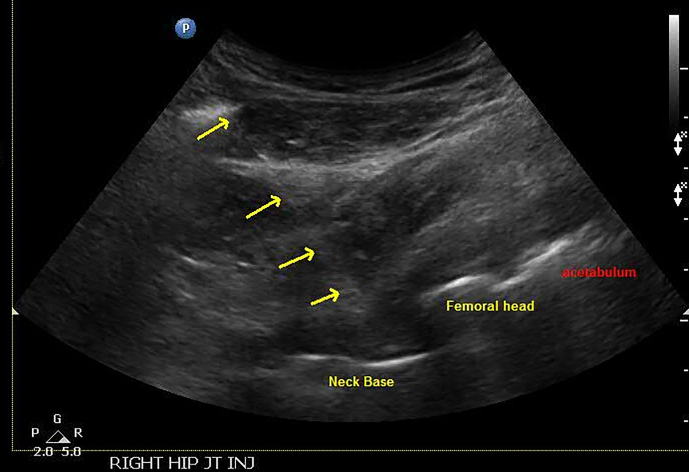
Ultrasound image with needle placement and labeled aspects of a total hip (neck, femoral head, and acetabulum).

On ultrasound, the femoral head and acetabulum were seen as two white lines proximal to the femoral neck. Depth of view was adjusted so that the prosthetic neck could be identified as the third straight white line (Fig. 3).

**Figure 4:**
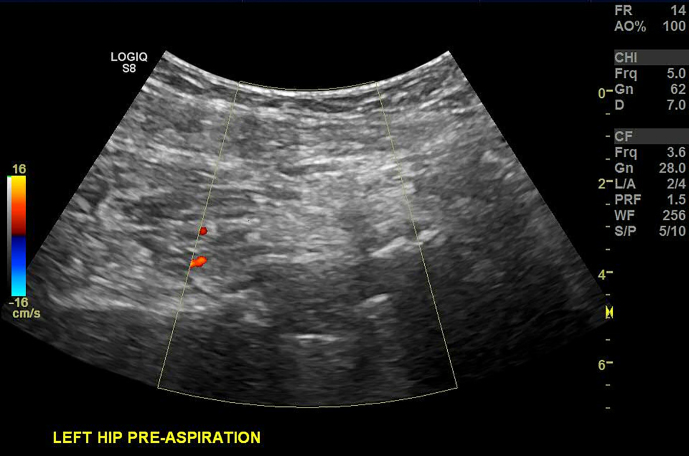
Ultrasound image of hip arthroplasty with color Doppler for identification of the circumflex artery.

Color Doppler was turned on for localization and avoidance of the circumflex artery and variant vessels (Fig. 4).

Once the neck of the prosthesis was identified on the ultrasound screen, the planned injection site and direction for needle placement was marked. Aspirations were performed with a 3.5 in. 18-gauge spinal needle or a 6 in. length in obese patients.

**Figure 5:**
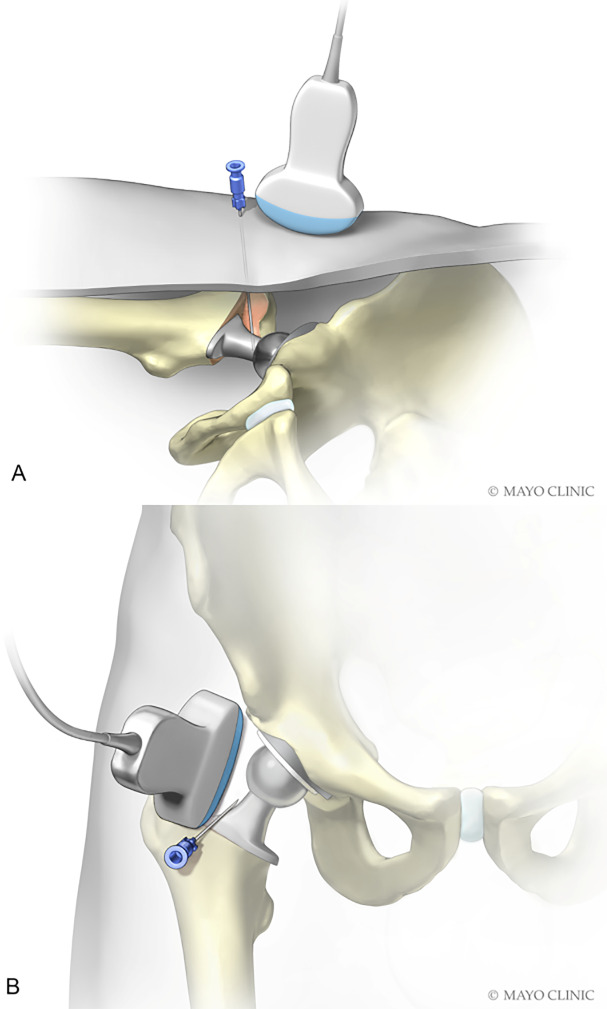
Depiction of needle placement lateral and slightly deep to neck of prosthesis. (Used with permission of Mayo Foundation for Medical Education and Research. All rights reserved.)

The skin was coated twice with separate ChloraPrep applicators. A wide antiseptic field was created to allow for sterile transducer movement (Lynch et al., 2019). Sterile towels were placed about the anterior hip (see Figs. 1 and 2). The skin was anesthetized with methylparaben-free 1 % lidocaine using a 25-gauge needle. Sterile gel was placed on the patient's skin. An 18-gauge spinal needle was slowly advanced while gradually injecting lidocaine for anesthesia and hydro-dissection to aid visualization of the needle path. The needle was aimed and advanced to the anterior neck of the prosthesis. Once the needle entered the capsule, and was at the neck of the prosthesis, no intentional additional lidocaine was injected to avoid dilution of the sample. The needle was left in place, and an aspiration was attempted with a 10 mL sterile syringe. If no fluid was obtained, the needle was redirected slightly laterally so that it could be felt advancing adjacent to the lateral aspect of the prosthetic neck. It was advanced posteriorly approximately 1–2 cm past the anterior aspect of the neck (Fig. 5a and b).

This was found to be the most reliable needle tip location for obtaining synovial fluid. As a final option, if no fluid was obtained, lavage was performed. Five to 10 mL of sterile non-bacteriostatic saline was injected. Aspiration was performed again and repeated at least once as necessary to obtain fluid. Ultrasound images were saved during the procedure. The aspiration syringe was detached, and then the needle was removed.

Fluid studies sent were as ordered by the treating orthopedic surgeon. If lavage was required, synovial leukocyte count and neutrophil percentage were not obtained due to dilution. For metal-on-metal bearings, manual cell counts were requested to prevent false elevation of leukocyte counts from metal particles (Wyles et al., 2013). The aerobic cultures were reported in 5 d, anaerobic cultures in 14 d, fungal cultures finalized at 24–30 d, and mycobacterial cultures at 42 d.

A scheduled aspiration was canceled or postponed in patients who presented with rash or skin changes at the intended skin puncture site due to risk of introducing an infection. A maximum serum international normalized ratio (INR) of 3 within 2 weeks prior to procedure was required for patients taking warfarin (Ahmed and Gertner, 2012). Aspirations were performed in patients taking oral anticoagulants (Yui et al., 2017). Antibiotics were discontinued for 2 weeks prior to aspiration; however, at the discretion of the ordering surgeon, an exception was made for patients on antibiotic suppression therapy for chronic infection.

## 4 Results

Between May 2016 and April 2019, 373 aspirations in 339 patients with hip arthroplasties were performed. Twenty-two patient records were not included since consent for review of records for research purposes had not been obtained. This resulted in 349 aspirations in 317 patients. Fourteen patients had bilateral aspirations and 21 had repeat aspirations. For sex distribution, 56 % were male. The right hip was aspirated in 55 % of cases. The distribution of amount of fluid obtained is given in Table 1.

**Table 1:** Fluid aspiration amounts with percentage of total aspirations and lavage percentage.

Aspiration amount obtained	Dry tap	0.2–1.0 mL	1.0–1.9 mL	2.0–3.9 mL	4.0–35 mL	>60 mL
N (total 349)	1	13	71	93	157	14
Percentage of total aspirations	0.03 %	4 %	20 %	26 %	45 %	4 %
Number requiring lavage (total 32)	1	0	11	9	11	0
Percentage of each group	100 %	0 %	15 %	10 %	7 %	0 %

The mean aspiration fluid volume was 17 mL with median volume of 4 mL. These values do not include hips requiring lavage, which was used in 9 %. Due to insufficient fluid, need for lavage with dilution of synovial fluid, or fluid too thick to determine cell count, 15 % of patients did not have cell count analysis performed.

Aspiration volumes were not affected by sex with both yielding a median aspiration amount of 3 mL. Table 2 shows volumes were varied for increased BMI, dislocated components at time of aspiration, patients with draining sinuses, and patients requiring lavage. Despite the variance in aspiration volume, none were statistically significant ( p value <0.05 ).

BMI had the highest correlation with median aspiration volumes but did not reach negative statistical significance ( p value). Patients with draining sinuses tended to have low aspiration fluid volumes despite the use of lavage 50 % of the time. The one dry tap was in a hip with a draining sinus in which lavage had been performed. Hip aspiration in patients who met ICM 2018 criteria for infection yielded higher average but lower median fluid amount.

**Table 2:** Fluid aspiration average (mean) and median for various patient, arthroplasty, and fluid characteristics and infected vs. non-infected as per ICM 2018 criteria.

	N (%)	Average (mean) (mL)	Median (mL)
All aspirations	349 (100 %)	15.7	3.0
Draining sinus ${}^{a}$	18 (5 %)	12.9	1.7
Dislocated prosthesis	10 (3 %)	14.0	10.0
Lavage used	32 (9 %)	2.8	2.0
Antibiotic cement	38 (11 %)	48.4 ${}^{b}$	3.5
BMI 17.5–29.7	152 (46 %)	24.9	3.0
BMI 30–39.9	134 (41 %)	9.2	3.0
BMI ≥40	42 (13 %)	2.8	2.0
ICM 2018 criteria “infected or surgeon treated as infected” (6 of 87) ${}^{c}$	87 (25 %)	28.4	2.8
ICM 2018 criteria “not infected, inconclusive, or insufficient” ${}^{c}$	262 (75 %)	11.9	4

${}^{a}$ 50 % of the aspirations performed in patients with a draining sinus required lavage. ${}^{b}$ Average increased due to aspirates of 1466 and 165 in two patients. ${}^{c}$ Infected aspirations required lavage (12 %, n=11 ). Non-infected, inconclusive, or insufficient required lavage (7 %, n=18 ). Surgeon treated 6 of the 87 cases as infected based on fresh frozen pathology demonstrating inflammation, periprosthetic fluid cloudiness, and/or gross wound tissue inflammation.

Adverse events are listed in Table 3. One patient on Eliquis had a bloody tap with bleeding at the injection site. He later presented to the emergency room, was diagnosed with hematoma, and eventually due to decreased hemoglobin was transfused 1 unit of packed red blood cells (PRBC).

**Table 3:** Adverse events with treatment.

Adverse event	Treatment	N
Pain at injection site	Ice and acetaminophen	1
Hematoma	Ice, pressure dressing	1
Hematoma in patient on Eliquis	Pressure dressing, elastic hip spica wrap, transfusion 1 unit PRBC for Hg drop from 10.5 to 7.8	1
Bleeding at injection site	Pressure dressing	1

Comparing hip aspiration culture results that were negative with patients who had revision total hip surgery and positive cultures, only one case developed PJI that was not previously known to be infected. That patient was on metronidazole until 4–5 d prior to the hip aspiration with surgical culture positive for *Staphylococcus epidermidis*. Table 4 lists all discordant negative hip aspiration results compared to revision hip surgical cultures.

**Table 4:** Hip aspiration cultures without growth of organism discordant with surgical cultures with at least one positive organism.

N	Specifics of surgical patient
16	Surgical culture result considered contaminant by surgeon and/or infectious disease consultants (see Table 9)
4	Known prior infections
2	Draining sinus present
3	Elevated serum erythrocyte sedimentation rate; elevated serum C-reactive protein; systemic symptoms; elevated synovial fluid differential leukocyte count; elevated percentage leukocytes from aspiration
1	Metronidazole discontinued 4 d before aspiration; *Staphylococcus epidermidis* infection found; surgery performed 10 weeks after aspiration
1	Known soft tissue infection hip; arthroplasty not infected

At completion of the study, 196 patients had revision of their aspirated hip joint.

Comparing surgical results of hip arthroplasty patients (infected and not infected) to ICM 2018 criteria for PJI obtained from hip aspiration and chart review, there was an 88 % accuracy of the ICM 2018 criteria. Accuracy, sensitivity, specificity, PPV, and NPV with 95 % confidence intervals of synovial fluid aspirate samples are listed in Table 5. A subset of the temporary metal spacers with antibiotic cement is included.

**Table 5:** Accuracy, sensitivity, specificity, PPV, and NPV of surgical results (infected or not infected) compared with ICM 2018 criteria derived from aspirate results or patient criteria; N=196 .

	Sensitivity	Specificity	PPV	NPV	Accuracy
Revised hip surgical results vs. ICM 2018 criteria	83 %	91 %	83 %	91 %	87 %
95 % confidence interval	71–91	84–95	71–91	84–95	
Temporary metal spacers with antibiotic cement surgical results vs. ICM 2018 criteria	100 %	75 %	71 %	100 %	85 %
95 % confidence interval	48–100	35–97	29–96	54–100	

Of the 349 aspirations, 196 had documented operative results. Surgical data were compiled from 1 July 2016 through to 29 February 2020.

The most common appearance of aspirated fluid was straw colored in 33 % (115). Sixty-nine patients (20 %) had bloody fluid aspirated. Thirty-nine percent of the aspirations in hips with draining sinuses were bloody. Lavage was utilized in thirty-two patients (9 %).

In Table 6, bloody aspirates and aspirates requiring lavage were compared to ICM 2018 criteria for validation that these compromised culture results yield helpful information to decide PJI probability.

**Table 6:** accuracy, sensitivity, specificity, PPV, and NPV of aspiration results to ICM 2018 criteria.

	Sensitivity	Specificity	PPV	NPV	Accuracy
Bloody aspirates vs. ICM 2018 ${}^{*}$ ( N=41 )	35 %	90 %	70 %	68 %	69 %
95 % confidence interval	15–59	74–98	35–93	52–82	
Lavage-required aspirates vs. ICM 2018 ${}^{*}$ ( N=15 )	36 %	100 %	100 %	44 %	59 %
95 % confidence interval	13–65	63–100	48–100	23–72	

${}^{*}$ Smaller sample sizes lead to larger confidence intervals.

Only 2 % (7) of the aspirations resulted in cultures deemed to be contaminants. Two of these hips had repeat aspirations performed which resulted in no growth of cultures and normal synovial fluid leukocyte counts and neutrophil percentages. The remaining five aspirations had a single colony growth, or growth was in broth only with a leukocyte count less than 2000 and neutrophil percentage under 60 %. Appendix A lists the contaminants and ultimate results of surgery.

Of the 349 aspirations, aerobic cultures were obtained in 99 % (348) and anaerobic cultures in 98 % (346). Positive culture results are listed in Table 7. One of the *Staphylococcus lugdunensis* was considered a contaminant, the other contaminants are indicated in the table.

**Table 7:** Microorganisms isolated from aspiration cultures (SCN represents staphylococcal negative).

Groups of microorganisms	Genus and species	N
*S. aureus*	*Staphylococcus aureus*	10
SCN	*Staphylococcus epidermidis*	12
	*Staphylococcus lugdunensis*	4
Enterococci	*Enterococcus faecalis*	4
Gram-negative bacilli	*Pseudomonas aeruginosa*	3
	*Escherichia coli*	2
	*Enterobacter cloacae* complex	1
	*Salmonella enterica*	1
Streptococci	*Streptococcus agalactiae*	3
	*Streptococcus mitis* group	1
Anaerobe	*Cutibacterium avidum*	3
	*Cutibacterium acnes*	2
	*Clostridium perfringens*	1
	*Finegoldia magna*	1
Fungi	*Candida albicans*	1
Other	*Bacillus circulans*	1
	*Bacillus licheniformis* ${}^{a}$	1
	*Gemella morbillorum*	1
	*Gordonia bronchialis*	1
	Gram-pos. bacillus resembling Paenibacillus sp. ${}^{a}$	1
	*Kocuria palustris* ${}^{a}$	1
	Large spore-forming aerobic Gram-positive bacillus ${}^{a}$	1
	*Micrococcus luteus* ${}^{a}$	1
	*Penicillium Sp.* ${}^{a}$	1

${}^{a}$ treated as contaminants

Table 8 lists aspiration types and matching culture percentages.

**Table 8:** Percentage of aspirate cultures that matched surgical cultures.

Aspirate characteristic	N	Percentage matched
All known surgical cases	196	81 %
Bloody aspirate	41	73 %
Lavage used	15 ${}^{a}$	60 %
Temporary metal spacers with antibiotic cement	14 ${}^{b}$	64 %

${}^{a}$ Five cultures had no growth, and four cultures grew identical organisms. ${}^{b}$ Six cultures had no growth, and five cultures grew identical organisms.

Finally, Table 9 lists the surgical culture results discordant with aspiration culture results.

**Table 9:** Discordant aspiration vs. surgical culture results.

Aspiration culture result	Surgical culture result	N
*Cutibacterium acnes*	*Cutibacterium acnes*	
	*Staphylococcus epidermidis*	
	*Staphylococcus warneri*	
*Enterococcus faecalis*	*Enterococcus faecalis*	
	*Staphylococcus epidermidis*	
*Enterococcus faecalis, Pseudomonas Aeruginosa,*	*Enterococcus faecalis*	
*Finegoldia magna*	*Finegoldia magna*	
	*Staphylococcus aureus*	
	*Enterococcus faecalis*	
	*Streptococcus mitis*	
*Staphylococcus epidermidis*	*Staphylococcus epidermidis*	
	*Cladosporium species*	
No growth	*Aerococcus sanguinicola, Pseudomonas aeruginosa*	1
	*Cutibacterium acnes* ${}^{*}$	2
	*Dermabacter hominis*	1
	*Enterococcus avium* (soft tissue infection)	1
	*Enterococcus faecalis*	1
	*Micrococcus flavus* ${}^{*}$ , *Cutibacterium acne* ${}^{*}$	1
	*Micrococcus luteus* ${}^{*}$	2
	*Mycobacterium bovis, Staphylococcus epidermidis* ${}^{*}$	1
	*Staphylococcus aureus*	3
	*Staphylococcus capitis* ${}^{*}$	1
	*Staphylococcus capitus* ${}^{*}$ , *Eggerthella lenta* ${}^{*}$	1
	*Staphylococcus epidermidis* ${}^{*}$	5
	*Staphylococcus epidermidis*	3
	*Staphylococcus hominis* ${}^{*}$	1
	*Staphylococcus saprophyticus*	1
	*Streptomyces* ${}^{*}$	1

${}^{*}$ considered contaminants by surgeon and/or infectious disease consultants

## 5 Discussion

This study demonstrates that meticulous ultrasound-guided aspirations performed in a clinic setting can yield sufficient fluid amounts that are useful and accurate when determining the presence of PJI in painful hip arthroplasties. The described technique with placement of the aspiration needle lateral to the femoral neck has not been described with ultrasound guidance but was previously diagramed for fluoroscopy (Brandser et al., 1997). The needle placement lateral to and past the femoral component neck is the most important aspect of the technique. Brandser et al. (1997) described placement past the femoral neck using fluoroscopy and reported similar success rate of aspiration, in 181 of 185 patients, with a dry tap rate of 2 %. The few articles in which use of ultrasound for aspiration of hip arthroplasties is presented do not describe our technique.

Craig (2013) discussed ultrasound examination of post-surgical hips, but the technique of aspiration was not presented. Eisler et al. (2001) reported on a lateral approach in 80 patients with ultrasound that yielded sparse aspiration results which was insufficient to obtain cultures 23 % of the time. Saline solution was not injected in the cases with a dry tap. In contrast, we obtained fluid for cultures in 97 % of the hips. In one series, dry tap rate was 13 %, and in 4 % of patients no fluid was aspirated even after 10 mL of saline solution was instilled into the joint (Taylor and Beggs, 1995). A recent publication based on landmarks for guidance (Li et al., 2021) had a dry tap rate of 45 %. Sconfienza et al. (2021) describe use of a Tru-Cut needle to take a biopsy for 37 % of aspiration attempts with dry taps. We were able to obtain at least 1 mL of fluid without lavage in 87 % (304 of 349) of the hips aspirated, avoiding the need for biopsy or repeat needle placement. With the additional use of lavage (9 % of the aspirations), we had only one dry tap.

The one study by Randelli (2018) comparing fluoroscopy with ultrasound did not describe ultrasound-specific technique nor indicate fluid volumes obtained. Ultrasound-guided aspiration was found to be more sensitive and specific compared to fluoroscopy. We had similar sensitivity and specificity.

Ultrasound guidance for painful hip aspirations has advantages for both patients and staff. In a study by Byrd et al. (2014), patients preferred hip injections performed with ultrasound to those performed with fluoroscopy. Ultrasound can detect fluid and does not expose staff or patient to radiation (Földes et al., 1992; van Holsbeeck et al., 1994). Disadvantages stem from difficulty visualizing patients with higher BMI and the need for ultrasound training to perform the procedure. Our findings demonstrated a trend toward lower overall fluid amounts in patients with higher BMI, but this did not reach statistical significance. Dislocated prostheses theoretically would be more difficult to visualize but yielded higher fluid aspiration amounts in our study.

A lower yield of fluid was obtained in patients with draining sinuses. Half ( n=18 ) required lavage and even with lavage one resulted in a dry tap. This is pertinent given a sinus track is a major determinant of PJI (Parvizi et al., 2011, 2018). Most articles do not mention draining sinuses in evaluation. Spangehl et al. (1999) allude to draining sinus as cause for 6 % (Parvizi et al., 2011) of 202 hip arthroplasty aspirations, yielding insufficient fluid for synovial white blood cell count analysis.

Recent research has indicated that aspirates diluted with either blood or saline were of poor quality (Deirmengian et al., 2020). Deirmengian et al. (2020) found sensitivity for poor- versus good-quality fluid samples were 69 % versus 97 % for synovial white blood cell count and 88 % versus 95 % for polymorphonuclear cell percentage (Deirmengian et al., 2020). Our results of fluid characterized as bloody show similar poor sensitivity, 35 % as per ICM 2018 criteria for PJI, with decreased accuracy of 79 vs. 89 % compared to all aspirate samples.

Despite this, a positive culture from bloody fluid was valuable with specificity of 93 %.

The use of lavage dilutes aspirate samples, so we did not send these for leukocyte count or neutrophil percentage. Few studies report on results when lavage is used. Li et al. (2019) reported on a landmark-guided approach which included aspiration of hips and knees. Forty-two percent of the hip aspirations required use of 10 mL of normal saline. Overall sensitivity of culture for hips and knees was 80 % with specificity of 96 %. In 2017 Newman et al. (2017) used saline lavage in 21 % of hips with antibiotic cement spacers. They found aspirations obtained with lavage to have 76 % accuracy. There was only one positive culture from 21 hips for 100 % specificity but 17 % sensitivity. Our fluid samples, including those from hips with antibiotic spacers, obtained with lavage positively matched culture results 60 % of the time and had an accuracy of 59 % compared with ICM 2018 guidelines. Lavage-obtained samples had 100 % specificity. So, if a lavage-required aspiration yields a positive culture, the patient most likely has PJI with that culture result.

A single culture due to an organism of relatively low virulence from synovial fluid or intraoperative culture was considered a contaminant. We had a low rate of aspirates: 2 % (Craig, 2013) deemed to be contaminated. Attempts to lessen the chance of contamination with skin flora in this study was performed by preparing the skin twice with 2 % chlorhexidine gluconate in 70 % isopropyl alcohol, use of sterile gloves, preparation of a sterile field large enough to allow for movement of the transducer, placement of sterile towels, and use of sterile ultrasound gel. Sherman et al. (2015) recommend sterile preparation of the entire injection field, because preparation of only 6 cm wide sterile field in a simulated study was associated with skin contamination, perhaps due to contact of transducer with nonsterile skin.

For limitations, this was a retrospective study; thus, we were unable to obtain information on duration of infection. Since the study was performed at a tertiary referral center, most infections were likely to be chronic. All internal and external medical records available were reviewed, but some patients may have had a subsequent revision at an outside institution that was not identified. Not all hip aspirations at our institution were included during the study period since patients who underwent fluoroscopic or ultrasound-guided aspirations in radiology department were not identified. Comparing fluoroscopic with ultrasound guidance would be a future study to pursue.

We were not able to verify if all patients were off antibiotics at the time of aspiration. It is the approach of orthopedic surgeons at our institution, however, to discontinue antibiotics in patients for at least 14 d prior to aspiration to allow for accurate culture results. An exception to discontinuing antibiotics occurred in patients with persistent symptoms of infection. In those patients, obtaining fluid to determine leukocyte count and neutrophil percentage was prioritized.

In conclusion, this study demonstrates a high-yield, safe, ultrasound-guided aspiration technique of hip arthroplasties performed in a clinic setting to aid in the diagnosis of PJI. Placement of the needle lateral and past the neck of the femoral component yields the best chance of obtaining fluid and avoiding dry taps. The use of lavage to obtain fluid yields helpful information when cultures are positive. Bloody aspirates are less accurate but have high specificity. Patients with draining sinuses have lower fluid yield and require lavage half of the time. With painstaking ultrasound-guided technique, sufficient synovial fluid with low contamination rate and 88 % accuracy is achieved for the diagnosis of periprosthetic hip infections.

## Data Availability

Data storage is with Mayo Clinic REDCap secure web platform (PID 65652). This is supported by grant UL1TR002377. Authors: Holly Duck and Suzanne Tanner. Separate analysis was performed with Excel. These data are available upon request. All data obtained from Mayo Clinic patients are confidential.

## Appendix A

**Table A1:** Contaminant organisms with subsequent treatment.

Contaminant organism	Repeat aspiration	Treatment	Culture results; surgical pathology	Post-surgical results
*Kocuria palustris* on first aspiration	yes; no growth	Two-stage arthroplasty	*Staphylococcus epidermidis* 4/5 surgical cultures; positive inflammation on pathology	7 months post-op second-stage infection free; deceased 8 months post-op
*Staphylococcus lugdunensis*	yes; no growth	Revision acetabulum with liner and femoral head exchange	4/4 surgical cultures negative; no inflammation on pathology	1 year post-op no complications or infection
*Bacillus licheniformis* broth only	no	Removal of greater trochanteric hardware	2/2 surgical cultures negative; no pathology sent; gross inspection per surgeon no infection	2 years post-op no complaints no infection
*Staphylococcus epidermidis*	no	One-stage exchange treated as if infected	4/4 cultures negative; surgical pathology; no acute inflammation	2 years post-op no infection
Bacillus Gram positive 1 colony	no	No surgery	NA	NA
*Micrococcus luteus* 1 colony	no	No surgery	NA	NA
Bacillus Gram positive	no	No surgery	NA	NA

NA: not available
